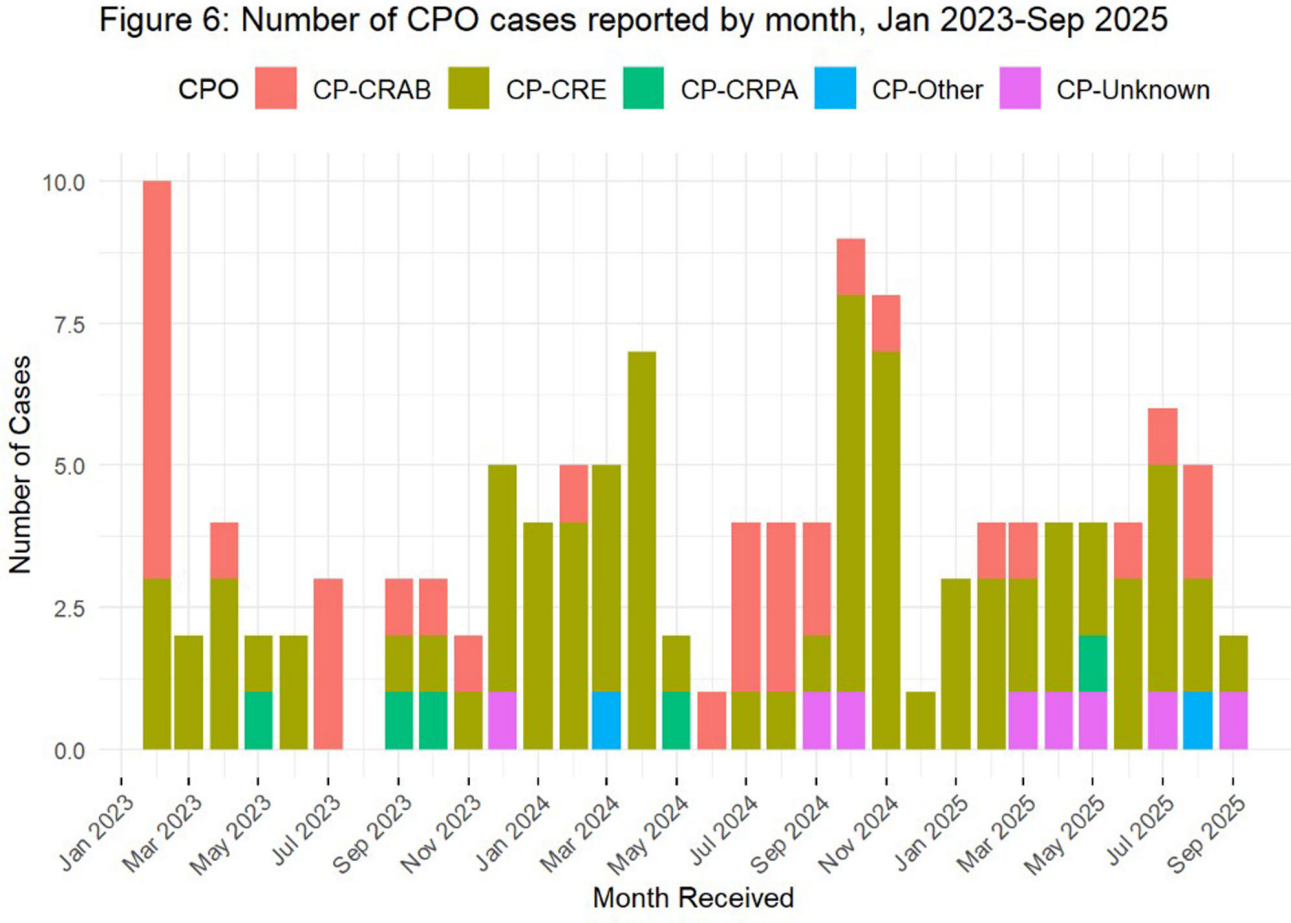# 291 Prevention of Secondary Cases after a Measles Exposure at an Academic Medical Center

**DOI:** 10.1017/ash.2026.10650

**Published:** 2026-06-23

**Authors:** Vani Nimbal, Joseph Clement, Lina Castro, Godfred Masinde, Farrell Tobolowsky

**Affiliations:** 1 San Francisco Department of Public Health; 2 SF Dept of Public Health

## Abstract

**Background:** Carbapenemase-producing (CP) organisms (CPOs), including Enterobacterales (CRE), Acinetobacter baumannii (CRAB), and Pseudomonas aeruginosa (CRPA), harbor carbapenemase genes that confer resistance and cause transmissible, difficult-to-treat infections, particularly in healthcare settings. In September 2022, California expanded reporting from CP-CREs to all CPOs. Through 2023, the state noted rising cases, multiple gene mechanisms, and rare combinations like NDM CRAB spreading statewide. However, recent data on all reportable CPOs remain limited. This analysis examines organism and gene distribution and annual trends in San Francisco (SF) following reporting expansion. **Methods:** We reviewed CPO cases, defined as isolates with a specific organism-carbapenemase gene combination, reported by SF healthcare facilities from January 1, 2023 to September 30, 2025. Clinical isolates were genetically characterized by local/state public health laboratories. Demographics, clinical, and epidemiological characteristics were analyzed. Crude incidence rates (IR) were calculated using SF population estimates, and temporal trends assessed using Poisson regression. **Result:** A total of 126 cases from 101 patients were included. Among these patients, median age was 64 years; 59 (58%) were male, and 68 (67%) resided outside SF. Of 126 cases, most were identified in short-term (70%) and long-term (18%) acute care hospitals (STACH and LTACH); 93% were healthcare-associated. CPO cases included 79 (63%) CP-CRE, 32 (25%) CP-CRAB, 5 (4%) CP-CRPA, and 10 (7.9%) unknown/other. Among the CP-CREs, Klebsiella pneumoniae (33%), Escherichia coli (32%), and Enterobacter cloacae (11%) predominated. Common specimen sources included urine (25%), respiratory (25%), and rectal (screening, 19%). CP-CREs were mainly associated with KPC (41%) and NDM (35%), while CP-CRAB frequently harbored OXA-variants (31%) and multiple mechanisms (31%, all NDM plus OXA-variant). Crude IR increased in 2024 to 6.3 cases/100,000 persons, driven by CP-CRE (4.5 cases/100,000 persons). NDM predominated in 2024 (2.4 cases/100,000 persons) before declining below KPC in 2025 (NDM: 1.3; KPC: 1.5 cases/100,000 persons). Incidence was highest among males and adults aged 65-79 years. No significant monthly trend was observed (IRR = 1.00, p = 0.74). **Conclusion:** Cases in SF increased from 2023 to 2024, with monthly trends through partial 2025 data suggesting stabilization. Our data showing 7% of cases with no/unknown healthcare-association align with estimates of 6-10% community-association from previous studies, reinforcing healthcare settings as the primary context for transmission. CP-CRE were predominantly KPC and NDM, consistent with recent reports; however, CP-CRAB showed greater multi-mechanistic resistance, with NDM/OXA-variant combinations as common as OXA-variants alone. Ongoing local surveillance and healthcare collaboration is critical to monitor evolving organism–gene patterns.

**Figure:**
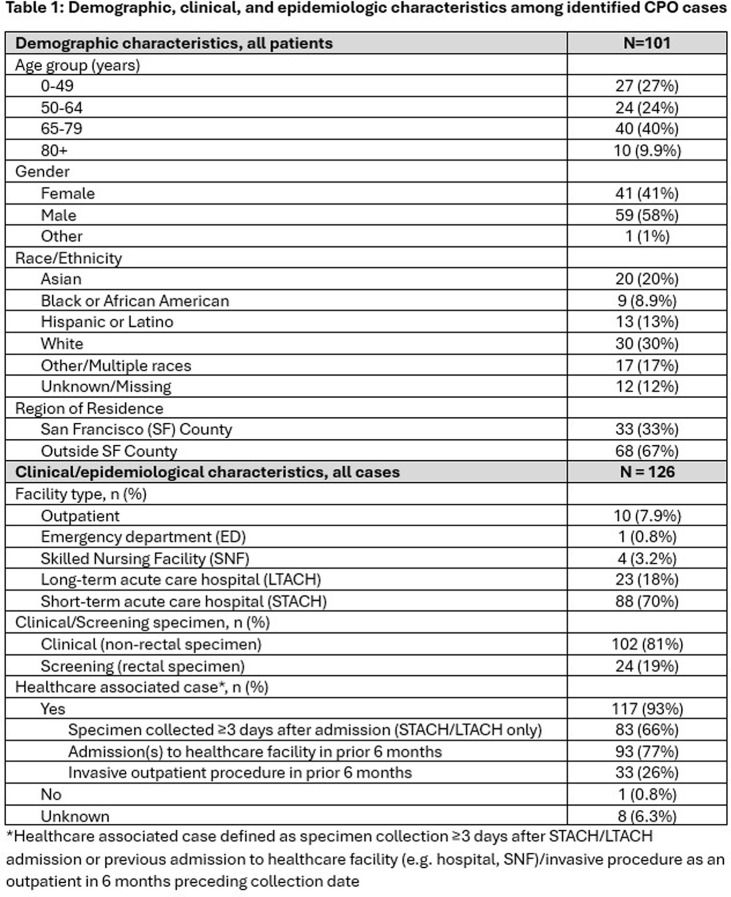

**Figure:**
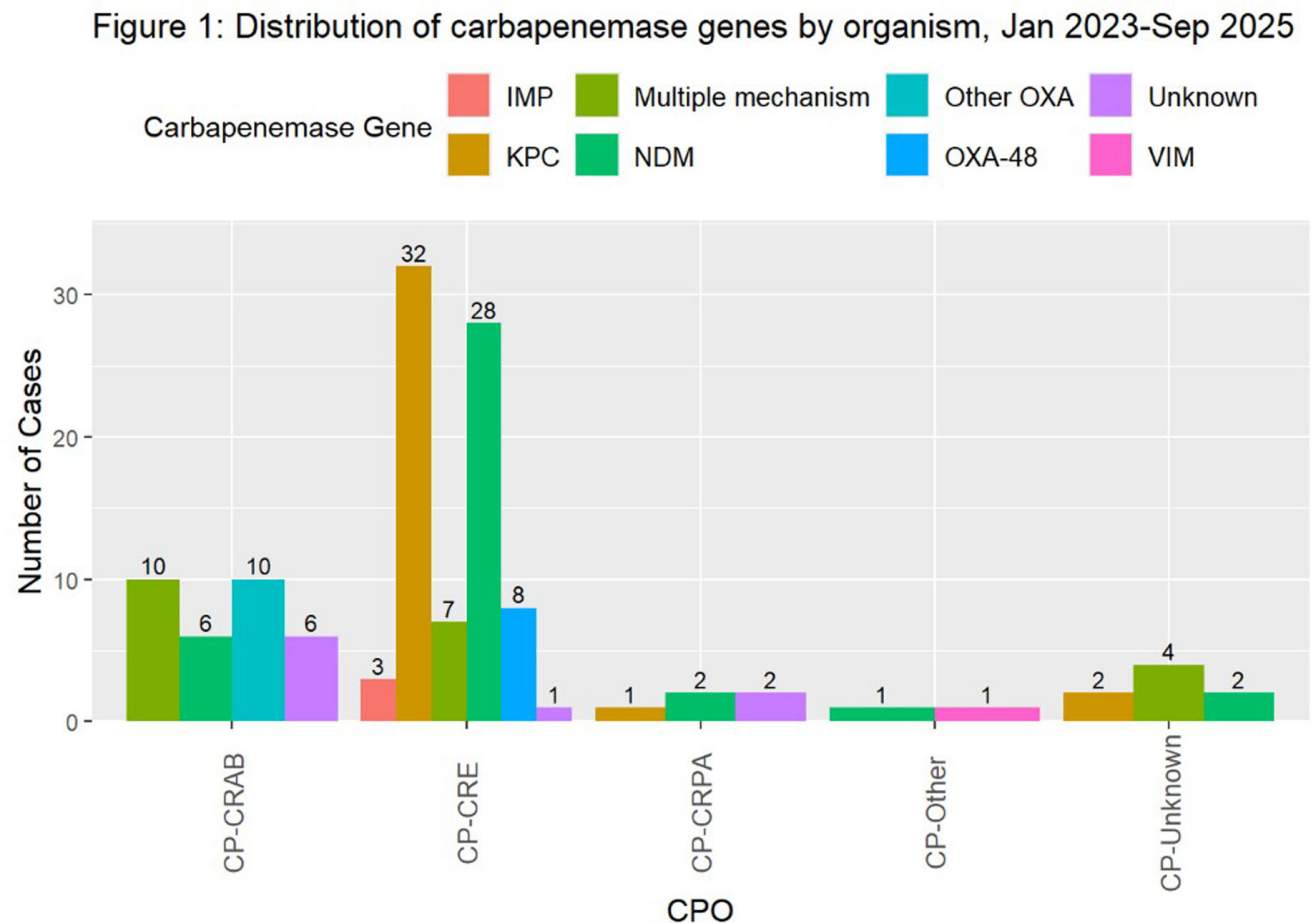

**Figure:**
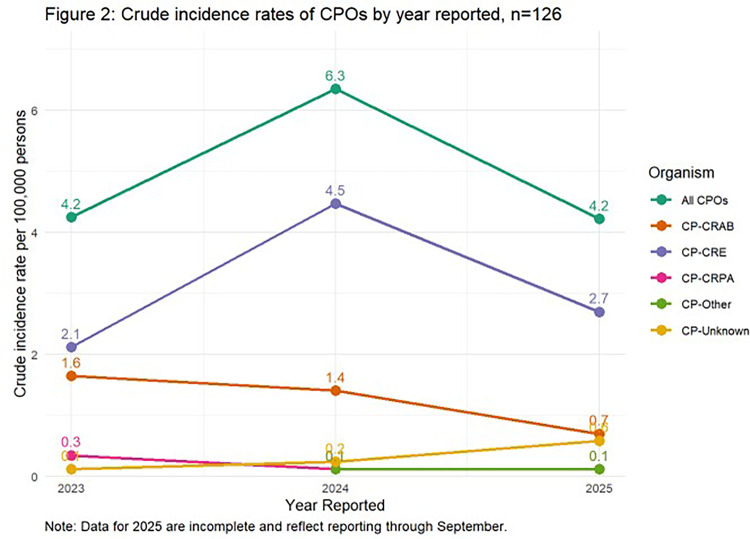

**Figure:**
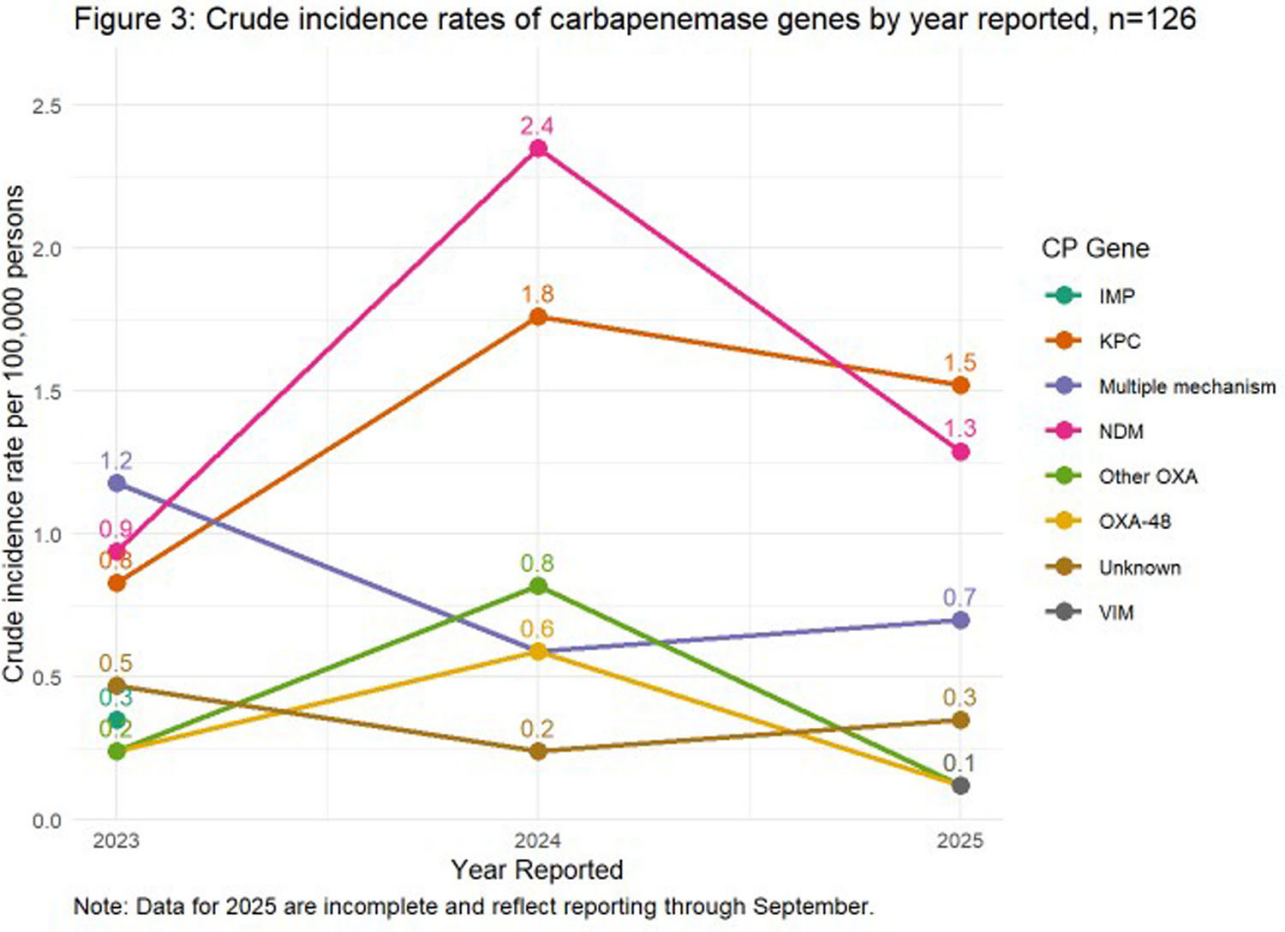

**Figure:**
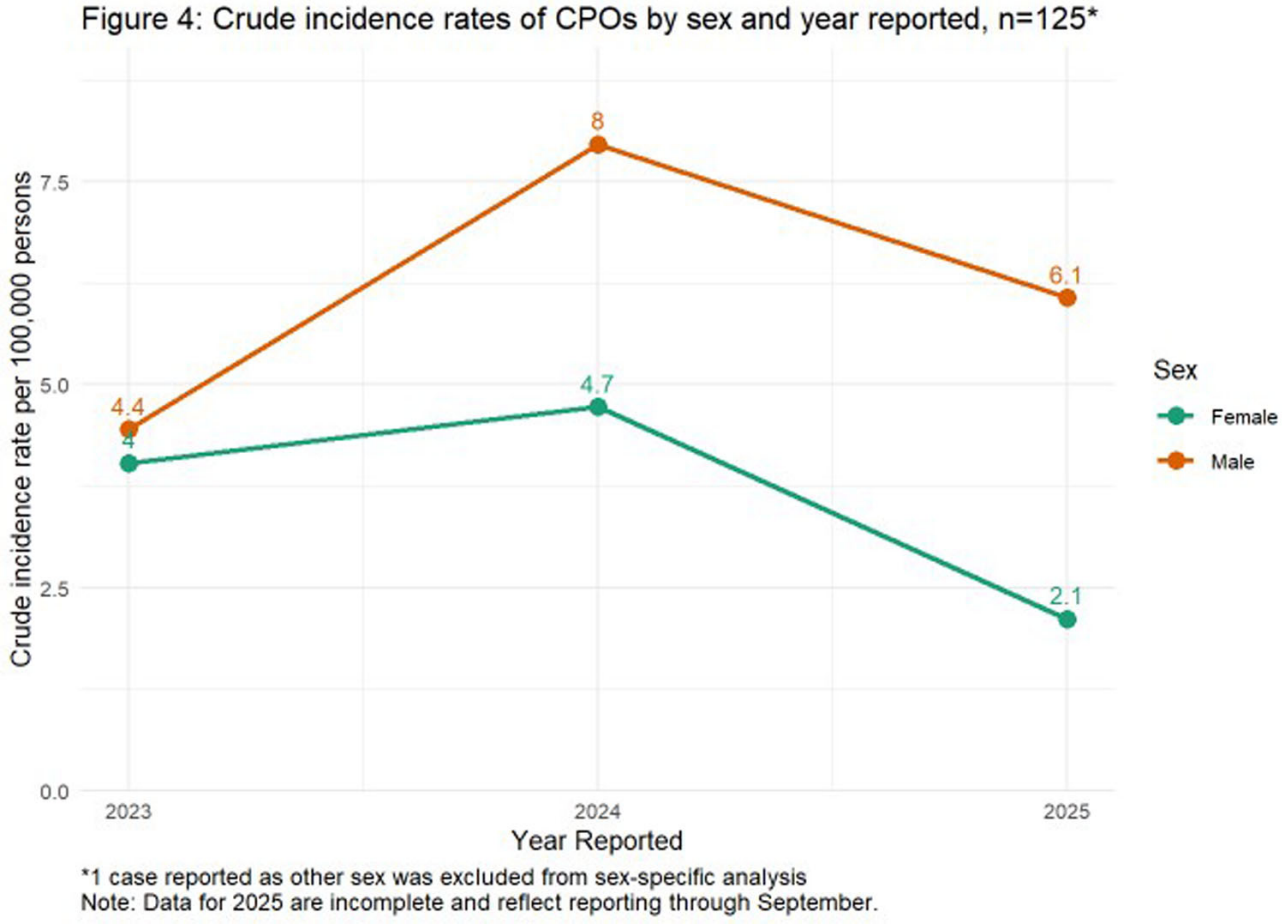

**Figure:**
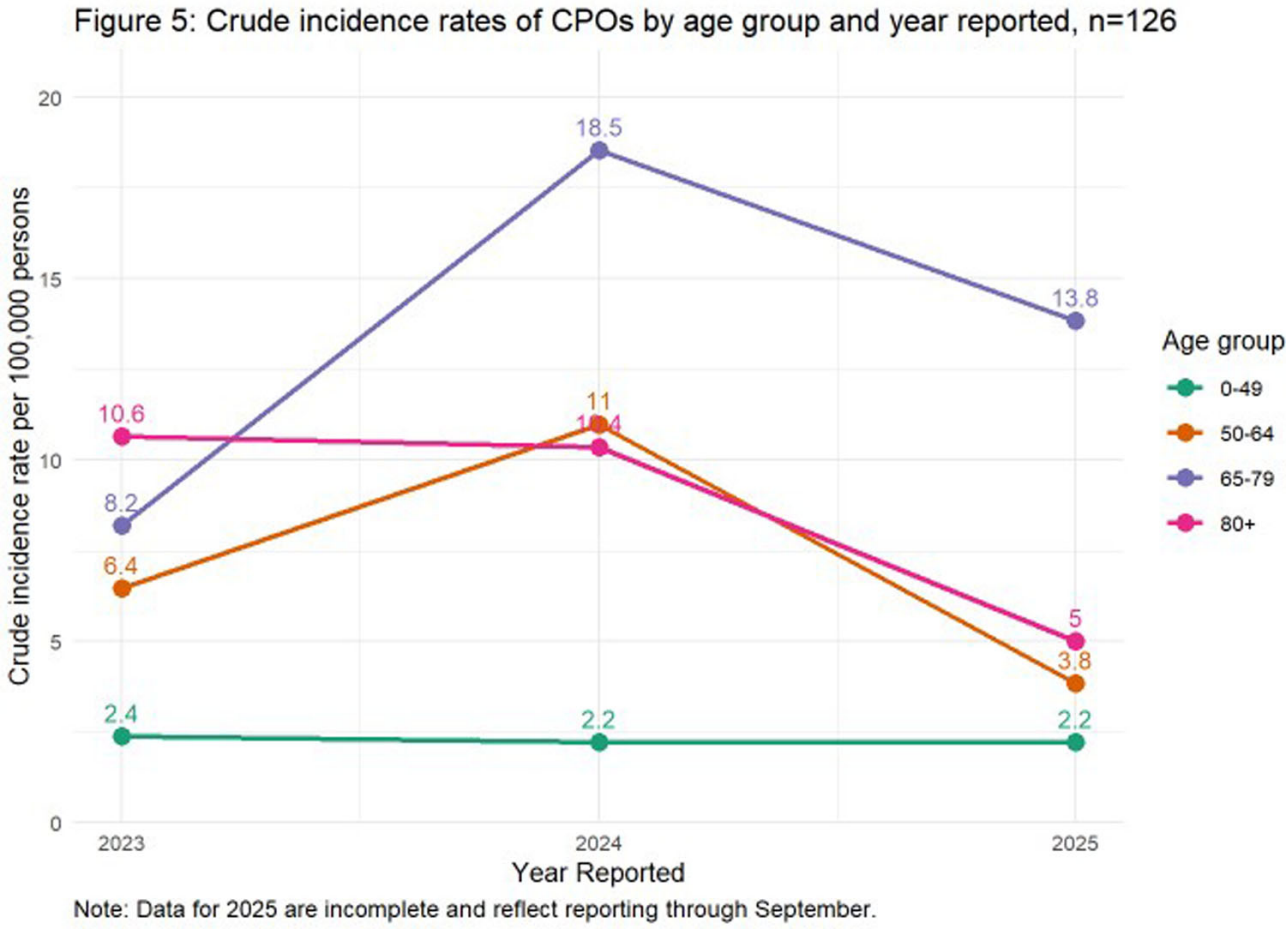

**Figure:**